# Self-supervised contrastive learning using CT images for PD-1/PD-L1 expression prediction in hepatocellular carcinoma

**DOI:** 10.3389/fonc.2023.1103521

**Published:** 2023-03-03

**Authors:** Tianshu Xie, Yi Wei, Lifeng Xu, Qian Li, Feng Che, Qing Xu, Xuan Cheng, Minghui Liu, Meiyi Yang, Xiaomin Wang, Feng Zhang, Bin Song, Ming Liu

**Affiliations:** ^1^ Yangtze Delta Region Institute (Quzhou), University of Electronic Science and Technology of China, Quzhou, China; ^2^ Department of Radiology, West China Hospital, Sichuan University, Chengdu, China; ^3^ The Quzhou Affiliated Hospital of Wenzhou Medical University, Quzhou People’s Hospital, Quzhou, China; ^4^ Institute of Clinical Pathology, West China Hospital, Sichuan University, Chengdu, China; ^5^ School of Computer Science and Engineering, University of Electronic Science and Technology of China, Chengdu, China; ^6^ Department of Radiology, Sanya People’s Hospital, Sanya, China

**Keywords:** hepatocellular carcinoma, PD-1/L1, deep learning, self-supervised learning, contrastive learning, computed tomography

## Abstract

**Background and purpose:**

Programmed cell death protein-1 (PD-1) and programmed cell death-ligand-1 (PD-L1) expression status, determined by immunohistochemistry (IHC) of specimens, can discriminate patients with hepatocellular carcinoma (HCC) who can derive the most benefits from immune checkpoint inhibitor (ICI) therapy. A non-invasive method of measuring PD-1/PD-L1 expression is urgently needed for clinical decision support.

**Materials and methods:**

We included a cohort of 87 patients with HCC from the West China Hospital and analyzed 3094 CT images to develop and validate our prediction model. We propose a novel deep learning-based predictor, Contrastive Learning Network (CLNet), which is trained with self-supervised contrastive learning to better extract deep representations of computed tomography (CT) images for the prediction of PD-1 and PD-L1 expression.

**Results:**

Our results show that CLNet exhibited an AUC of 86.56% for PD-1 expression and an AUC of 83.93% for PD-L1 expression, outperforming other deep learning and machine learning models.

**Conclusions:**

We demonstrated that a non-invasive deep learning-based model trained with self-supervised contrastive learning could accurately predict the PD-1 and PD-L1 expression status, and might assist the precision treatment of patients withHCC, in particular the use of immune checkpoint inhibitors.

## 1 Introduction

Immune checkpoint inhibitors (ICIs) have emerged as potentially effective treatments for patients with 26 hepatocellular carcinoma (HCC) in the advanced stage and is moving fast because of the encouraging clinical results in regulating T-cell activation and proliferation (1–4). Programmed cell death protein-1 (PD-1) and programmed cell death-ligand-1 (PD-L1) are members of the widely studied immune checkpoint pathway. PD-1 is mainly expressed on the membrane of T cells and acts as a negative regulator of the antigenic response (5). PD-L1 inhibits cytotoxic activity of T cells by binding to PD-1 expressed on their surface, and allows the immune escape of tumor cells (6). ICIs that work by blocking PD-1 and PD-L1 checkpoints (7–10) have shown potential for the improved treatment of patients with HCC. However, only a few patients with HCC benefit from this immunotherapy, and the durable response rate to anti-PD-1 treatment is only 15–20% (11, 12). Thus, it is important to be able to identify HCC patients who would benefit the most from blocking the PD-1/PD-L1 pathway.

Previous studies have demonstrated that the expression status of PD-L1 in tumors is associated with clinical outcomes and treatment responses to PD-1/PD-L1 pathway inhibition (13–15). Therefore, evaluation of PD-1 and PD-L1 expression is crucial for identifying individuals who will respond to checkpoint blockade, precise treatment decision-making, and prognostic improvement in patients. Histopathological examination is the gold standard for evaluating the expression of PD-1 and PD-L1, but histopathological biopsies are invasive procedures, and are associated with the risk of sampling errors and morbidity. An alternative non-invasive method for measuring PD-1/PD-L1 expression is urgently needed for clinical decision support.

More recently, attention has focused on the fast-growing deep learning (DL) field, which has made great achievements in many practical applications such as image recognition and classification (16–23). Based on sufficient training data, DL can effectively manage a large amount of high-dimensional and noisy data by capturing typical complex features and nonlinear relationships. An increasing number of DL technologies have been applied in medical-related research (24–28). For instance, Zhang et al. established a diagnostic tool based on a DL framework for screening common, treatable blinding retinal diseases. Apostolopoulos et al. (29) utilized a convolutional neural network (CNN) to predict the presence of Covid-19 using X-rays and achieved promising results. Wang et al. (30) proposed a DL framework for analyzing whole-slide lymph node images to identify lymph nodes and tumour regions. Besides, a series of works have utilized machine learning (ML) or DL on the gene mutation or immune checkpoint pathway prediction in HCC or intrahepatic cholangiocarcinoma (ICC) (31–33). Similarly, applying DL to the prediction of PD-1/PD-L1 expression using computed tomography (CT) images could enable effective, non-invasive prediction, and promote individual and precise treatment decision-making for patients with HCC.

However, several challenges that differ from traditional computer vision tasks are encountered when applying DL to predict PD-1 and PD-L1 expression using CT images. The first is how to extract proper deep representation from CT images. The expression of PD-1 and PD-L1 is not directly reflected in CT images, which requires the model to extract more accurate deep representations from CT images. The second problem is the data utilization efficiency. As some patients may have complete CT images but incomplete clinical information like PD-1 or PD-L1 expression. These CT images were excluded during data processing due to incomplete clinical information, which significantly reduced data volume and data utilization efficiency. The effective use of these unlabeled CT images is worthy of further exploration. Finally, there is the problem of simultaneous prediction of PD-1 and PD-L1 expression. PD-1 and PD-L1 levels are strongly correlated, and predicting the expression of the two metrics independently would not only increase the computational cost of the network and reduce the computational efficiency, but may also ignore the correlation between the expression of the two proteins. It is worth exploring a suitable network structure to predict the expression of PD-1 and PD-L1 simultaneously. We have attempted to address these challenges in our study.

**Figure 1:**
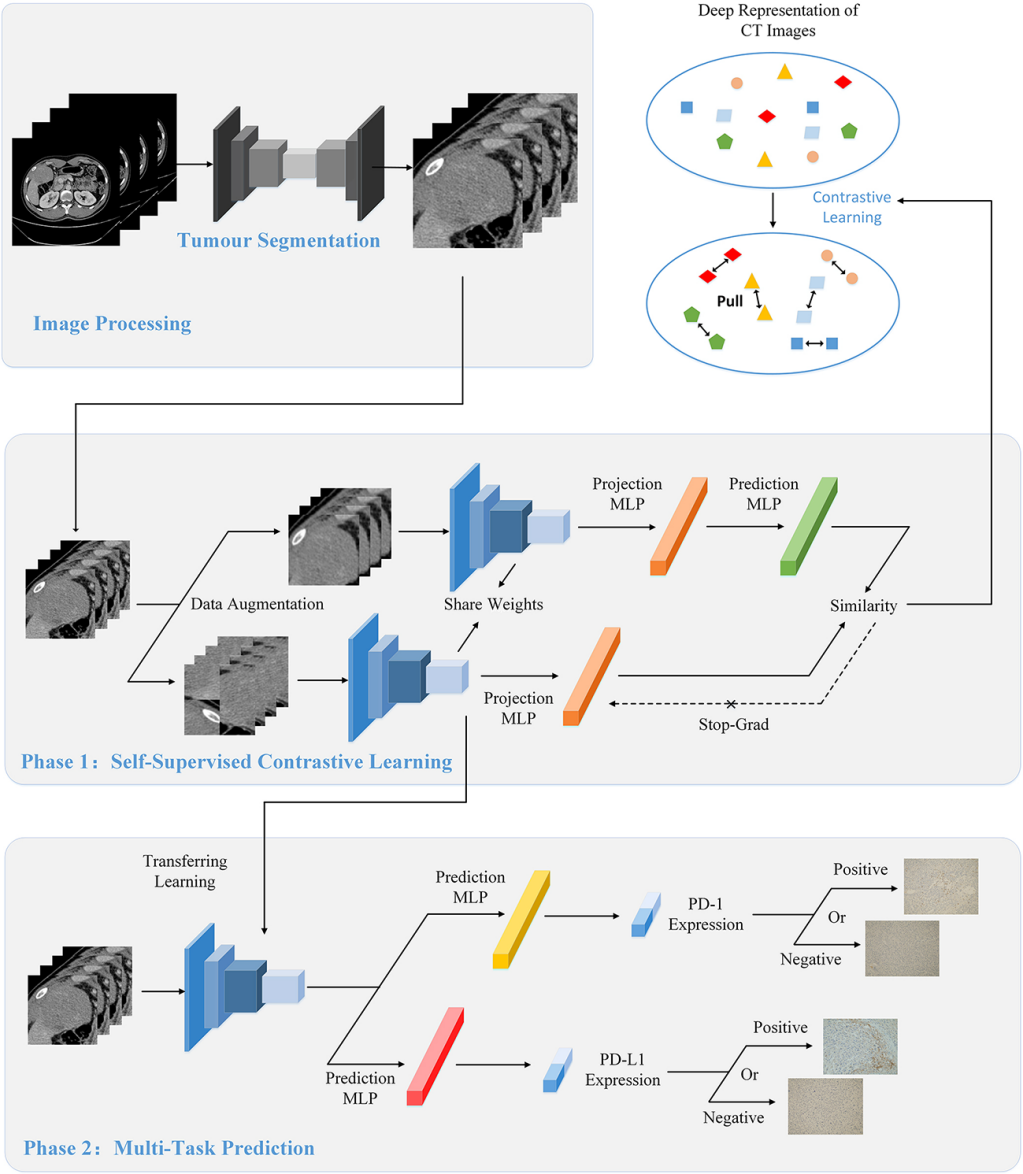
The overall flow of image processing, self-supervised contrastive learning, and multi-task prediction. (Top) During the image processing stage, we extracted the tumor regions from the original CT images as the input of the model using the segmentation network. (Middle) We used contrastive learning to pre-train the proposed CLNet. A series of data augmentation strategies were used to produce two different views of the same image, and contrastive learning would improve the model’s ability by gathering the two different views. Besides, we added additional unlabeled CT images to the train set to increase the data patterns and improve the generalization of the model. (Bottom) We applied the model pre-trained with contrastive learning as the backbone model and used the transfer learning strategy to train the prediction model. Two independent Multilayer Perceptrons (MLP) were added for predicting PD-1 and PD-L1 expressions respectively.

In this study, we propose a novel DL-based model, Contrastive Learning Network (CLNet), for the noninvasive prediction of PD-1/PD-L1 expression status in HCC patients based on CT images. As shown in **Figure 1**, this prediction model was composed of the deep convolution model ResNet-50 (34) and two independent fully connected layers to simultaneously obtain the expression of PD-1 and PD-L1. To better extract deep representations, we applied self-supervised contrastive learning (35) in the first stage of development. In addition, we added a new data augmentation patch shuffle to enhance the learning of the local features. To improve the data utilization efficiency and enlarge the patterns of training images, we introduced unlabeled images that should have been discarded during data processing for self-supervised training. We recruited 87 of patients with HCC from the West China Hospital and analyzed 3094 CT images to develop and validate the prediction model. The experimental results show that our CLNet exhibited an AUC of 86.56% for PD-1 expression and an AUC of 83.93% for PD-L1 expression, outperforming other DL models and machine learning strategies.

## 2 Materials and methods

### 2.1 Patients

**Figure 2:**
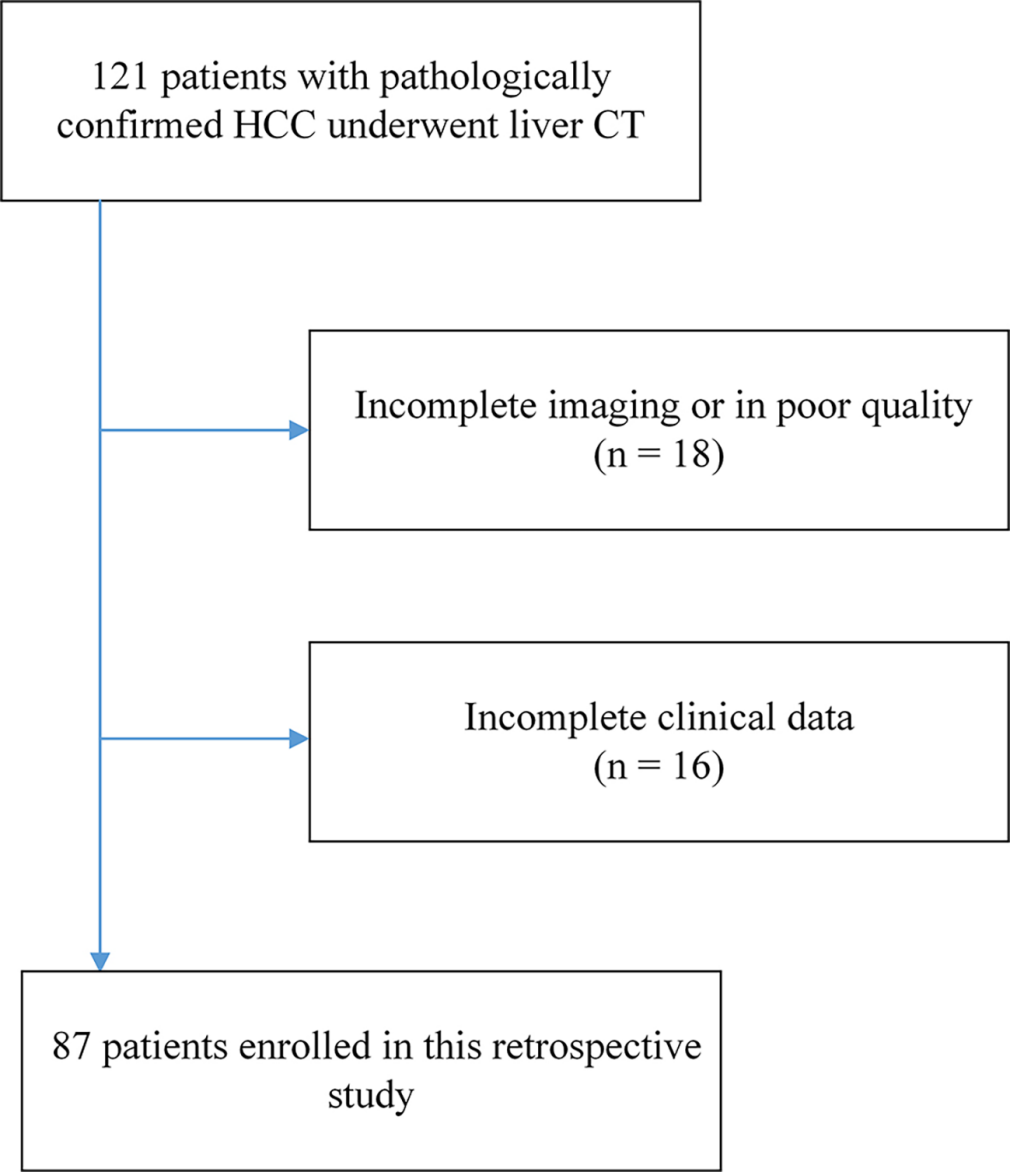
The flowchart of patient enrollment. A total of 87 patients with HCC from 121 were finally enrolled in this study.

The Institutional Review Board of West China Hospital approved our retrospective study. Due to our study’s retrospective nature, the requirement for written informed consent was waived. Patients in West China Hospital with a pathological diagnosis of HCC from July 2012 to October 2016 were retrospectively included in our study with the following inclusion criteria: (1) ages were 18 years older; (2) pathologically confirmed HCC; (3) the interval between contrast-enhanced CT imaging and surgery less than four weeks; (4) no history of preoperative treatment. The exclusion criteria were as follows: (1) Incomplete or poor-quality CT images (n = 18); (2) Incomplete clinical data (n = 16). A total of 87 patients with HCC were finally enrolled in this study as shown in **Figure 2**. Besides, we included 929 CT images from above 16 HCC patients with incomplete clinical data as auxiliary training images in the self-supervised training process, and the detailed illustration can be seen in the SubSection Model Development. Based on time domain, patients underwent surgery between July 2012 and September 2015 (n = 63) were assigned to training cohort, and the subsequent patients underwent surgery between October 2015 and October 2016 (n = 24) were assigned to validation cohort.

### 2.2 Image acquisition

CT images in this study were acquired from multi detector CT. All patients used three stages of CT (unenhanced phase, arterial phase, portal vein phase) with LightSpeed VCT (GE Healthcare) or Sensation 64 CT (Siemens Healthcare), and were injected with contrast type iopromide injection (iodine concentration, 300-370 mg/mL; volume, 1.5–2.0 ml/kg of body weight; contrast type, iopromide injection, Bayer Pharma AG). The arterial phase and the portal vein phase was obtained 25 seconds and 60 seconds after the injection of contrast agent. After obtaining the portal vein phase CT images, we chose the tumor region as the input of the network for prediction. To get precise tumor region, the tumor segmentation was performed on the initial CT images with SEVB-Net, which is a modified V-net developed by United Imaging Intelligence (36, 37). We centered the center point of the tumor region and expanded the 128×128 area as the final input.

### 2.3 Immunohistochemistry of PD-1 and PD-L1 expression

The paraffin tissue from surgically resected specimens were cut into 4 *µ*m-thick sections, dewaxed, hydrated, and then antigen retrieval. Then, tissue slides were incubated with primary antibodies using rabbit anti-human PD-1 polyclonal antibody (5 *mu*g/ml, cat # PA5-20351; Invitrogen; Thermo Fisher Scientific, Inc., Waltham, MA, USA.) and anti-human PD-L1 monoclonal antibody (5 *µ*g/ml cat # 14-5983-82; Invitrogen) at 4°C overnight, followed by incubation with secondary antibody (cat # K5007; Dako). PD-1 staining was performed with 3,3’-diaminobenzidine and counterstained with hematoxylin. Result of PD-1 expression was presented as the proportion of PD-1 + tumor infiltrating immune cells (PD-1 + immune cells/total immune cells), and the cut-off values for PD-1 overexpression were determined by x-tile software, cases with expression greater than 5% were considered as PD-1-positive. The number of PD-L1 cells was quantified at ×400 (0.0484 mm^2^), results of PD-L1 expression were presented as the proportion of PD-L1 + tumor cells (PD-L1 + tumor cells/total tumor cells), and the cut-off values for PD-L1 overexpression were determined to be 3%.

### 2.4 Self-supervised contrastive learning

In recent years, self-supervised learning has attracted many researchers’ attention in the field of DL. By using self-defined pseudo labels as supervision, self-supervised learning is able to decrease the cost of producing the labels of the dataset. Specifically, contrastive learning is one of the representative self-supervised learning methods (38–41), which gathers two perspectives of the same image (positive pairs) and rejects different images (negative pairs). Constructing two perspectives of the same image is to make close the model outputs of these two views and further makes the model learn the deep feature provided by the image itself. The model trained by contrastive learning can be applied to various downstream tasks because it has learned the deep feature from the image itself.

We used self-supervised contrastive learning (35) to train the network during the first training stage. The ability to extract a deep representation of images is one of the most important functions of the DL model, and the quality of the deep representation produced by the DL model is directly related to the final prediction performance. For our classification task, the expressions of PD-1/PD-L1 are not directly reflected in the CT images, it needs the model to have the ability to extract the deep feature from images. Contrastive learning is a novel self-supervised training strategy that can effectively mine deep representations of images. And the model trained by contrastive learning has learned from the image itself, so its ability of feature extraction is suitable for this task. Besides, contrastive learning does not need label information when extracting features, so the model trained by contrastive learning is also applicable to the expression prediction of PD-1 and PD-L1 at the same time. In this study, we adopted an advanced contrastive learning method, SimSiam (35), which utilizes only positive pairs. As shown in **Figure 1**, for a given CT input image *x*, different data augmentation methods are used to generate two different views *x*_1_ and *x*_2_ of *x*. ResNet-50 (34) followed by projection MLPs, was chosen as the backbone encoder *f*(*x*), and a prediction MLP head *h*(*x*) was added to one branch to match another view of the image. *x*_1_ and *x*_2_ entered two branches of the above models, and the outputs were obtained as follows:


(1) $$p_{1}≜h\left(f\left(x_{1}\right)\right)$$

(2) $$z_{2}≜f\left(x_{2}\right)$$

After obtaining the two output vectors, we use the negative cosine similarity to maximize the similarity between *x*_1_ and *x*_2_:


(3) $$D\left(p_{1},z_{2}\right)=-\frac{p_{1}}{\|p_{1}{\|}_{2}}\cdot \frac{z_{2}}{\|z_{2}{\|}_{2}}$$

where ||·||_2_ denotes the *l*2-norm. To prevent model collapse, a stop-gradient operation was performed on D(*p*_1_, *stopgrad*(*z*_2_)), and the final training loss was defined as follows:


(4) $$L=\frac{1}{2}D\left(p_{1},stopgrad\left(z_{2}\right)\right)+\frac{1}{2}D\left(p_{2},stopgrad\left(z_{1}\right)\right)$$

**Figure 3:**
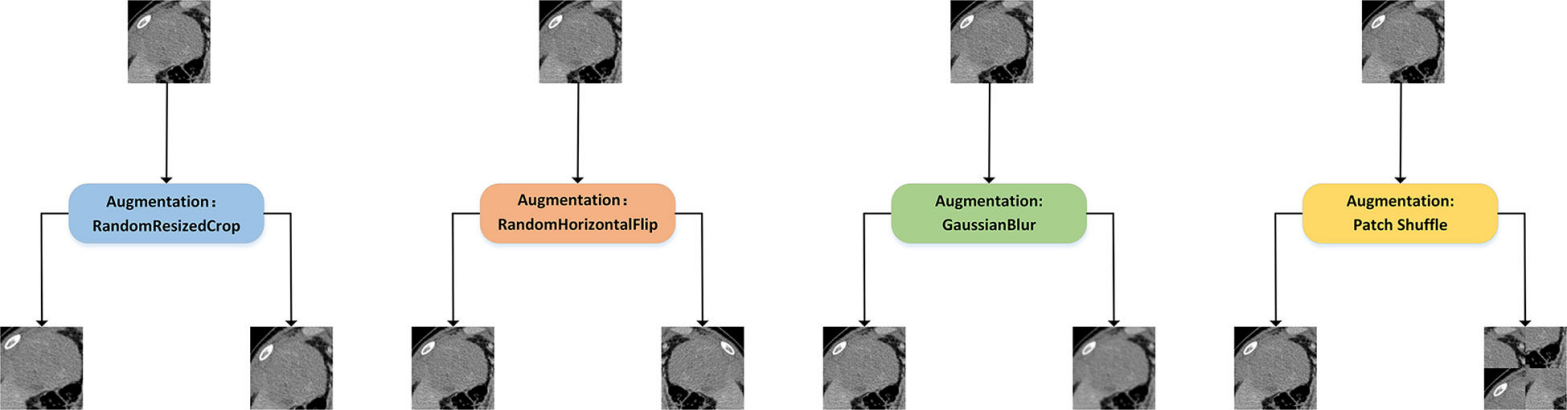
Illustration of different data augmentation strategies for contrastive learning. Random Resized Crop would crop a random part of the image and resize it to a uniform size. Random Horizontal Flip would randomly flip the image horizontally. GaussianBlur would randomly blur the image with Gaussian noise. In addition, we introduced the patch shuffle strategy that divided the image into four patches and randomly put the four patches together to deepen the model’s understanding of the local representation.

Constructing two different views of the same image is an important part of extracting the deep representation of CT images. We retained the commonly used data augmentation methods of contrastive learning, such as Random Resized Crop, Random Horizontal Flip, and ColorJitter. At the same time, we add a new data augmentation method, patch shuffle, for comparison. This data augmentation method divides the image into four patches and randomly puts the four patches together into one image with the probability of *P*. The training with contrastive learning increases the similarity between the patch shuffled image’s deep representation and the original image’s deep representation. This new data augmentation method aims to deepen the model’s understanding of the local representation of the training images. An illustration of the aforementioned data augmentation strategies is shown in **Figure 3**.

### 2.5 Multi-task prediction

The ability to determine PD-1 and PD-L1 expression is critical for personalized medical treatment and selection of immunosuppressive agents for patients with HCC. Relying on CT images of patients to simultaneously predict their PD-1 and PD-L1 expression is a meaningful but challenging task. In essence, these are two two-class classification tasks, i.e., judging whether the patient’s PD-1 expression is negative or positive and whether PD-L1 expression is negative or positive through the patient’s CTimage. The model trained by self-supervised contrastive learning is well suited for the simultaneous prediction of PD-1 and PD-L1 expression because it does not use PD-1 or PD-L1 labels but rather extracts tumor-related features from the CT images, which are generalizable to the prediction of both PD-1 and PD-L1 expression. Therefore, this model can be used as the backbone for multi-task prediction of PD-1 and PD-L1 expression. As shown in **Figure 1**, we applied the transfer learning strategy to train the prediction model in the second training stage. Specifically, we only retained the convolution layers of the contrastive-learning model and added two new fully connected networks for predicting PD-1 and PD-L1 expression. The two fully connected networks have the same structure, which is combined with three linear layers with batch normalization (BN) layers and ReLU activation functions. For labelled datasets, $D=\left\{x_{i},y_{i}^{1},y_{i}^{2}\right\}$ , *x_i_* denotes the training image, $y_{i}^{1}$ denotes the PD-1 expression label, and $y_{i}^{2}$ denotes the PD-L1 expression label. The convolution layers of CLNet are defined as *C*(·), the fully connected network for PD-1 prediction is defined as *F^PD^* ^-1^(·), and the fully connected network for PD-L1 prediction is defined as *F^PD^* ^-^ *^L^* ^1^(·). The output of fully connected network for PD-1 prediction *a^PD^* ^-1^ is computed as:


(5) $$a^{PD-1}=F^{PD-1}\left[C\left(x\right)\right]$$

Similarly, the output of fully connected network for PD-L1 prediction *a^PD^* ^-1^ is computed as:


(6) $$a^{PD-L1}=F^{PD-L1}\left[C\left(x\right)\right]$$

The predicted probability of PD-1 expression *p^PD^* ^-1^ for class *i* is computed by softmax as:


(7) $$p_{i}^{PD-1}=\frac{exp\left(a_{i}^{PD-1}\right)}{\sum_{j=1}^{C}exp\left(a_{j}^{PD-1}\right)}$$

And the predicted probability of PD-L1 expression *p^PD^* ^-^ *^L^* ^1^ for class *i* is computed by softmax as:


(8) $$p_{i}^{PD-L1}=\frac{exp\left(a_{i}^{PD-L1}\right)}{\sum_{j=1}^{C}exp\left(a_{j}^{PD-L1}\right)}$$

where *C* denotes the number of ground truth labels, i.e., positive and negative. In summary, the loss function of the prediction model consists of the cross-entropy of two classifiers for PD-1 and PD-L1 expression, which can be written as:


(9) $$loss=\frac{1}{2}[CrossEntropy\left(p^{PD-1},y^{1})+CrossEntropy\left(p^{PD-L1},y^{2}\right)\right]$$

With the above formula, we can update the network weights using the correspondence between the training images and PD-1/PD-L1 labels at the same time to achieve the effect of multi-task training. This not only improves the training efficiency of the network, but also helps the model to explore the relationship between PD-1 and PD-L1 expression as multi-task training.

### 2.6 Model development

The development of the model was divided into two stages. The first stage was self-supervised training, for which we used contrastive learning for better deep representation extraction. The second stage was the supervised training, for which we used transfer learning for the simultaneous prediction of both PD-1 and PD-L1 expression. In the first phase of training, we used unlabeled images to pre-train the model. A number of medical images with incomplete clinical information were added in actual image processing. Even though these images met the standard for network training, they would have been discarded owing to missing clinical information like PD-1 or PD-L1 status, which would have resulted in the waste of data resources. However, we were still able to include these images during the phase of self-supervised training. We collected additional 929 unlabeled CT images from 16 patients with a pathological diagnosis of HCC and incomplete clinical information in West China Hospital from July 2012 to October 2016. We included these unlabeled CT images in the training set for the self-supervised training stage. This not only improved the data utilization efficiency, but also increased the data patterns and improved the generalization of the model. Different from the training setting of supervised learning, the setting of the large epoch is beneficial for contrastive learning. The training epoch of contrastive learning was set to 800 because large training epochs can help improve the model’s ability to extract deep representations. The initial learning rate of contrastive learning is set to 0.025 with batch size 128. In the second phase of training, we followed the common practice of transfer learning by freezing the parameter value in the convolution layers of ResNet-50, and only training two fully connected modules. The training epoch of transfer learning was set to 100, and the initial learning rate is set to 0.5 with batch size 256. The learning rate in transfer learning was set to 0.5 because we found the learning rate in transfer learning often needed a higher value for better network performance than supervised learning. Besides, we chose the Stochastic Gradient Descent (SGD) optimizer to optimize the model training with momentum of 0.9. To the supervised DL models including Res-Net-50, VGGNet-19_BN, DenseNet-100, and PrymidNet-110, the training epoch was set to 200 with the initial learning 0.01, weight decay 0.001 and batch size 256, which was the optimal setting for a fair comparison. Python (3.7.13) was used to conduct this research on the Ubuntu (20.04.4) operating system. All experiments related to DL were performed using the Pytoch framework (1.11.0) with four NVIDIA GeForce RTX 3080ti GPUs.

### 2.7 Validation and statistics analysis

After obtaining the trained prediction model, we tested its performance on the validation set. There are a series of CT images from each patient in the validation set, and every CT image would separately enter into the prediction model to get its probability of PD-1 and PD-L1 expression. For each patient, we selected only the top *k* probability among all images and derive its mean value as the final probability of PD-1 or PD-L1 expression for this patient. Further, if the number of patient’s CT images is less than *k*, we would derive the mean value of all images’ probability as the final probability. The value of *k* was set to 10 for both PD-1 and PD-L1 prediction. We used this statistical approach because the most clearly expressed CT image often best reflect the tumor condition, and combining the expression of these images could provide a more reasonable prediction.

We used the area under the curve (AUC) values under the receiver operating characteristic (ROC) curve to assess the prediction performance of the proposed model for discriminating PD-1 and PD-L1 expression. Besides, accuracy (Acc), sensitivity (Sen), specificity (Spec) and Matthews correlation coefficient (Mcc) were also calculated for validation, as follows:


(10) $$Accuracy=\frac{TP+TN}{TP+TN+FP+FN}$$

(11) $$Sensitivity=\frac{TP}{TP+FN}$$

(12) $$Specificity=\frac{TN}{TN+FP}$$

(13) $$MCC=\frac{TP\times TN-FP\times FN}{\sqrt{\left(TP+FN\right)\left(TP+FP\right)\left(TN+FN\right)\left(TN+FP\right)}}$$

where TP, TN, FP, and FN represented the true positive, true negative, false positive, and false negative, respectively.

## 3 Results

### 3.1 Clinical characteristics

We retrospectively identified a cohort of 121 patients with histologically proven HCC between July 2012 to October 2016. Of the 121 patients, 87 who met the inclusion criteria were included and 34 patients were excluded. We assigned consecutive patients who underwent surgery between July 2012 to September 2015 to the training cohort (number: 63; average age: 53.13 ± 11.79 years; 57 men and 6 women) and patients who underwent surgery from October 2015 to October 2016 to the validation cohort (number: 24;average age: 47.96 ± 13.76 years; 22 men and 2 women). The validation cohort included younger patients relative to the training cohort. There were no sex differences between the training and validation cohorts. The training and validation cohorts had a prevalence of PD-1 positivity by immunohistochemistry of 41.27% and 41.67%, respectively, and the prevalence of PD-L1 positivity by immunohistochemistry was 22.22% and 29.17%, respectively. The baseline characteristics of patients in the training and validation cohorts are shown in **Table 1**. The *p*-value in **Table 1** describes the difference of the clinical and pathological characteristics of patients between the training and validation cohorts. The values (ALL P > 0.05) demonstrated that there was no statistical difference in the baseline characteristics and laboratory features between training and validation cohorts.

**Table 1:** The clinical characteristics of patients with HCC in the training and validation cohorts.

Patient characteristic	Development cohort	Validation cohort	*p*-Value
Number	63	24	–
Age, mean ± SD, yr	53.13 ± 11.79	47.96 ± 13.76	0.085
Gender			0.866
Male	57 (90.47)	22 (91.67)	
Female	6 (9.53)	2 (8.33)	
HBV			0.487
+	58 (92.06)	22 (91.67)	
-	5 (7.94)	2 (8.33)	
ALT (IU/L)			0.150
<40	38 (60.32)	11 (45.83)	
AST (IU/L)			0.078
<35	29 (46.03)	6 (25.00)	
ALB (g/L)			0.410
<40	17 (26.98)	6 (25.00)	
AFP (ng/mL)			0.421
<400	37 (58.73)	12 (50.00)	
CEA (ng/mL)			0.369
<3.4	38 (60.32)	16 (66.67)	
GGT (*µ*/L)			0.756
<45	23 (36.51)	7 (29.17)	
PLT (×10^9^/L)			0.335
<100	15 (23.81)	6 (25.00)	
TBIL (*µ*mol/L)			0.597
<17.1	36 (57.14)	15 (62.50)	
MVI			0.119
Absent	38 (60.32)	7 (29.17)	
Present	25 (39.68)	17 (70.83)	
BCLC			0.249
0-A	9 (14.29)	6 (25.00)	
B	33 (52.38)	12 (75.00)	
C	21 (33.33)	6 (25.00)	

### 3.2 Comparison with deep learning models

We first compared the CLNet model with other DL models in predicting PD-1/PD-L1 expression in a validation cohort. The other DL models included ResNet-50 (34), VggNet-19 BN, DenseNet-100 and PyramidNet-101 (42–44). These DL models were supervised models trained from beginning. We used grid search to find the optimal parameter setting under different training strategies, i.e., the optimal performance of our CLNet and other supervised DL models were provided for a fair comparison. As shown in **Table 2**, CLNet exhibited an AUC of 86.56% 83.93%, ACC of 84.38% and 83.33%, sensitivity (Sen) of 92.86% and 85.00%, specificity (Spec) of 80.88% and 82.14%, and MCC of 0.688 and 0.671 for the prediction of PD-1 and PD-L1 expression, respectively. Compared with ResNet-50, which has the same convolution structure as our model, the AUC for PD-1 and PD-L1 expression was increased by 8.41% and 8.57%, and the ACC was increased by 4.17% 266 and 6.25%, respectively. This illustrates that contrastive learning is conducive to the extraction of features related to PD-1 and PD-L1 expression. In addition, the proposed CLNet performs better than other models, such as DensNet-100 and PrymidNet-100, which illustrates the effectiveness of our model.

**Table 2:** Performance comparison between proposed CLNet with other DL models such as ResNet-50, VGGNet-19 BN, DenseNet-100, and PrymidNet-110 for predicting PD-1 and PD-L1 expressions.

Pathway	Model	AUC (%)	Acc (%)	Sen (%)	Spec (%)	Mcc
PD-1	ResNet-50	78.15	80.21	78.57	80.88	0.568
	VGGNet-19_BN	75.42	73.96	78.57	72.06	0.473
	DenseNet-100	78.36	81.25	78.57	**82.35**	0.592
	PrymidNet-110	75.63	76.04	75.00	76.47	0.483
	CLNet (ours)	**86.56**	**84.38**	**92.86**	80.88	**0.688**
PD-L1	ResNet-50	75.36	77.08	70.00	82.14	0.541
	VGGNet-19_BN	71.70	71.88	57.50	82.14	0.426
	DenseNet-100	73.75	73.96	**87.50**	64.29	0.519
	PrymidNet-110	72.86	73.96	82.50	67.86	0.520
	CLNet (ours)	**83.93**	**83.33**	85.00	**82.14**	**0.671**

AUC, Area under the receiver operating characteristic curve; Acc, Accuracy; Sen, Sensitivity; Spec, Specificity; Mcc, Matthews correlation coefficient. The highest value is in bold.

**Figure 4:**
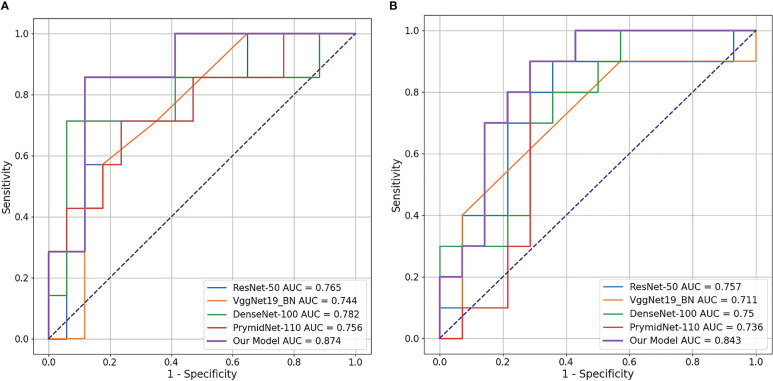
Receiver operating characteristic (ROC) curve of the four DL models and proposed CLNet. Our CLNet achieves the best performance in both PD-1 and PD-L1 prediction tasks. **(A)** denotes PD-1 expression and **(B)** denotes PD-L1 expression.

**Figure 5:**
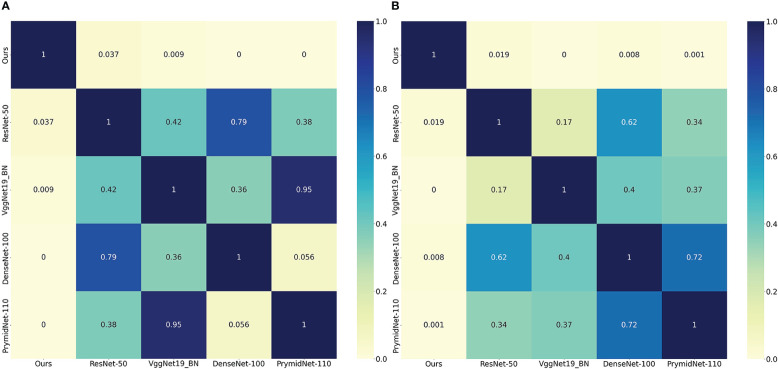
Heat map of the significance t-test, the values in each square of the heat map represent the corresponding p-values between two DL models, and two statistical variables were considered statistically significant when the p-value was less than 0.05. All statistical tests were performed on the predicted results of the validation cohort. The left heat map represents the corresponding p-values of PD-1 expression and the right represents PD-L1 expression. We set the value in each square to zero if the corresponding p-value is less than 0.001. **(A)** denotes PD-1 expression and **(B)** denotes PD-L1 expression.

We also used a receiver operating characteristic (ROC) curve to visualise the prediction results. **Figure 4** shows the comparison of ROC curves among the DL models. CLNet obtained the best area under the curve for PD-1/PD-L1 expression prediction. We also used the heat map to plot the results of the t-test to visualise the prediction differences of the above models. The values in each square of the heat map represent the corresponding p-values between two DL models, and two statistical variables were considered statistically significant when the p-value was less than 0.05. As shown in **Figure 5**, all p-values between our model and the other models were less than 0.05, which indicated significant differences in quality of predictions between CLNet and the other DL models.

### 3.3 Comparison with machine learning methods

In this subsection, CLNet was compared with other machine learning (ML) methods¸ (45) including K-nearest neighbor (KNN), support vector machine (SVM), decision tree. As shown in **Table 3**, the performance of our model was much better than that of traditional ML methods. The AUC of CLNet was 18.8% and 21.07% higher than that of the decision tree in predicting PD-1 and PD-L1, respectively. To explore the model’s ability to autonomously extract features, we did not introduce an additional feature extraction method when performing ML methods, similar to the development of CLNet. The results illustrate that our model has a stronger ability to predict PD-1 and PD-L1 expression than traditional ML methods.

**Table 3:** Performance comparison between our proposed model and traditional machine learning strategies for predicting PD-1 and PD-L1 expressions.

Pathway	Model	AUC (%)	Acc (%)	Sen (%)	Spec (%)	Mcc
PD-1	KNN	61.76	69.79	85.00	44.64	0.332
	SVM	66.17	69.79	82.14	64.69	0.465
	DecisionTree	67.76	59.38	**96.43**	44.12	0.397
	CLNet (ours)	**86.56**	**84.38**	92.86	**80.88**	**0.688**
PD-L1	KNN	58.30	60.42	60.00	60.71	0.238
	SVM	57.68	62.50	70.00	57.15	0.275
	DecisionTree	62.86	61.46	85.00	44.64	0.331
	CLNet (ours)	**83.93**	**83.33**	**85.00**	**82.14**	**0.671**

The highest value is in bold.

### 3.4 Ablation study

In this subsection, we describe several ablation experiments conducted to analyze the effectiveness of the proposed training strategy. We first compared the performance of the model trained only with normal training images during the self-supervised training phase, and the models trained by additional CT images of patients with incomplete clinical information but high CT image quality. **Table 4** shows that including more images in the self-supervised pre-training process could significantly improve the network’s performance in PD-1 and PD-L1 expression prediction (PD-1: p-value = 0.015; PD-L1: p-value = 0.013). While these unlabeled images are usually discarded during traditional data processing, we reused them for self-supervised pre-training because contrastive learning does not require label information. Experimental results prove that additional image information can extend the image patterns that the model learns, thus improving the model’s ability to extract image features.

**Table 4:** Performance comparison between training with and without extra unlabeled CT images.

Pathway	Model	AUC (%)	Acc (%)	Sen (%)	Spec (%)	Mcc
PD-1	CLNet_original	82.35	77.08	92.86	70.59	0.586
	CLNet (ours)	**86.56**	**84.38**	**92.86**	**80.88**	**0.688**
PD-L1	CLNet_original	78.21	75.00	**87.50**	66.07	0.547
	CLNet (ours)	**83.93**	**83.33**	85.00	**82.14**	**0.671**

CLNet original denotes the proposed model trained without additional images. The highest value is in bold.

To validate the effectiveness of the patch shuffle strategy used in contrastive learning, we conducted a group of comparative experiments in which we trained the network with and without the patch shuffle strategy. The results are summarized in **Table 5**. The results showed that adding the patch shuffle strategy improved the AUC and ACC by 6.50% and 8.34% for PD-1 expression and improved the AUC and ACC by 5.36% and 6.25% for PD-L1 expression compared with regular contrastive learning. The performance of training with only the patch shuffle strategy was not satisfactory, only achieving 72.06% AUC and 73.96% ACC on PD-1 and 71.25% AUC and 72.92% ACC on PD-L1. This shows that patch shuffle is an appropriate method for adding more image patterns during contrastive learning, but traditional data augmentation methods such as Random Resized Crop and Random Horizontal Flip are necessary. Further, we validated the performance of the patch shuffle strategy on supervised training based on ResNet-50. Results in **Table 6** show that patch shuffle augmentation does not improve the model ability in supervised learning, we consider the reason may be that the pattern provided by patch shuffle is benefit for capturing the invariance between the original image and the image shuffled on the patch unit during contrastive learning, but is difficult to be contained by supervised learning mode.

**Table 5:** Performance comparison among different data augmentation strategies for contrastive learning.

Pathway	Model	AUC (%)	Acc (%)	Sen (%)	Spec (%)	Mcc
PD-1	Original_aug	80.06	76.04	**96.43**	67.65	0.585
	Patch shuffle	72.06	73.96	78.57	72.06	0.482
	CLNet (ours)	**86.56**	**84.38**	92.86	**80.88**	**0.688**
PD-L1	Original_aug	78.57	77.08	77.50	76.79	0.554
	Patch shuffle	71.25	72.92	75.00	71.43	0.506
	CLNet (ours)	**83.93**	**83.33**	**85.00**	**82.14**	**0.671**

Original aug denotes the regulate data augmentation strategies used in contrastive learning. The highest value is in bold.

**Table 6:** Performance comparison between the supervised ResNet-50 with and without patch shuffle strategy.

Pathway	Model	AUC (%)	Acc (%)	Sen (%)	Spec (%)	Mcc
PD-1	ResNet-50	**78.15**	**80.21**	**78.57**	**80.88**	**0.568**
	+ patch shuffle	77.73	75.00	71.43	76.47	0.450
PD-L1	ResNet-50	**75.36**	**77.08**	**70.00**	**82.14**	**0.541**
	+ patch shuffle	73.57	70.83	60.00	78.57	0.393

Results show that patch shuffle cannot improve the performance of supervised training. The highest value is in bold.

Besides, we further explored the performance of the transformer-based model in supervised learning. Swin Transformer (46) was chosen because it needed less data to train compared with other transformer models. **Table 7** shows that Swin Transformer achieved a similar performance in AUC and ACC with ResNet-50, and higher Sen and lower Spec compared with ResNet-50. We would like to explore the performance of Swin transformer by modifying its modules in future work.

**Table 7:** Performance comparison among supervised ResNet-50, supervised Swin Transformer and our self-supervised CLNet.

Pathway	Model	AUC (%)	Acc (%)	Sen (%)	Spec (%)	Mcc
PD-1	ResNet-50	78.15	80.21	78.57	80.88	0.568
	Swin Transformer	80.67	79.17	85.71	76.47	0.573
	CLNet (ours)	**86.56**	**84.38**	**92.86**	**80.88**	**0.688**
PD-L1	ResNet-50	75.36	77.08	70.00	82.14	0.541
	Swin Transformer	75.00	75.00	**90.00**	64.29	0.543
	CLNet (ours)	**83.93**	**83.33**	85.00	**82.14**	**0.671**

The highest value is in bold.

For the settings of linear layers, the modules of BN and ReLU could help the model mitigate the impact of overfitting and are commonly used in linear layers. The reason that we chose the three layers is that there is a huge gap between the dimension of the linear layer’s input (2048) and the dimension of output (2). The three layers structure performs better than one and two layers by +5.04% and +1.68% of AUC in PD-1 prediction and +3.22% and +1.08% of AUC in PD-L1 prediction.

### 3.5 Visualization of deep representations

**Figure 6:**
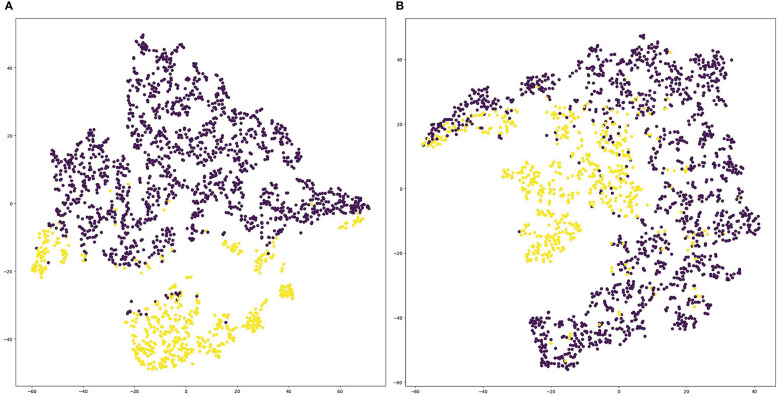
Visualization of deep representations from proposed model using t-SNE. Yellow dots represent the distribution of deep representations for positive cases, and purple dots represent the distribution of deep representations for negative cases. **(A)** denotes PD-1 expression and **(B)** denotes PD-L1 expression.

We visualized the deep representations of the proposed model using t-SNE, which could reduce the features of the high dimension into the low dimension. We used yellow dots to represent the distribution of positive cases’ deep representations, and purple dots to represent the distribution of negative cases’ deep representations. As shown in **Figure 6**, the deep representations of positive cases and negative cases for both PD-1 and PD-L1 expression are obviously separated by our CLNet. It proves that the model we proposed can effectively extract the deep features of CT images, and improve the discrimination ability for PD-1 and PD-L1 expressions.

## 4 Discussion

PD-1 and PD-L1 expression status assessed by immunohistochemistry can provide guidance for clinical decision-making and individualized treatment of patients with HCC. However, invasive needle biopsies for immunohistochemistry may cause sampling errors and morbidity; therefore, an alternative non-invasive method to predict PD-1 and PD-L1 is urgently needed. In this study, we built and validated the first DL model based on self-supervised contrastive learning using CT images to non-invasively predict the expression of PD-1 and PD-L1 in HCC patients. We introduced a self-supervised pre-training approach to reuse the unlabeled images that should have been discarded in supervised model training, enriching the model’s input patterns and improving the data utilization efficiency. We also improved the original contrastive learning method by adding a comparison between a normal image and a patch-shuffled image with a certain probability, which increased the difficulty of model training, thus improving the capability of the prediction model to capture deep representations. Ablation studies showed the effectiveness of our proposed strategies in improving the data utilization efficiency and enhancing the learning ability of the model. Our model was compared with different DL and ML models, and our AUC value and other major metrics were the highest among them for the prediction of PD-1 and PD-L1 expression in HCC patients. Specifically, our proposed model can non-invasively predict the expression status of PD-1 and PD-L1 in HCC patients, and achieved a performance of 86.56% AUC, 84.38% ACC, 92.86% Sen, 80.88% Spec, 0.688 Mcc for PD-1, and 83.93% AUC, 83.33% ACC, 85.00% Sen, 82.14% Spec, and 0.671 MCC for PD-L1. The results indicated that our model could achieve reliable predictive performance, which also validated the correlation between CT images of HCC patients and expression status of the immune checkpoint pathway. The experimental results also show the superiority of our approach compared to traditional DL and ML models. This improvement proves that applying contrastive learning to the DL model with additional CT images and various data patterns is beneficial for model training.

The model proposed in this study has a number of advantages over existing models. First, as a discriminative approach, contrastive learning groups similar examples closer and diverse examples farther from each other to learn the underlying representations of images (38). A series of relevant studies have focused on this basic idea and improved the contrastive strategy by modifying the data augmentation, model structure, loss function, and memory bank (39, 40, 47–49). We applied the self-supervised contrastive learning strategy SimSiam (35) to improve the ability of the DL model to extract deep representations of CT images. We chose SimSiam with ResNet-50 as the basic architecture to obtain a better deep representation. Compared with other contrastive learning strategies, our training strategy maintained its promising performance by grouping similar examples closer without diverting examples far from each other, which could significantly reduce the training overhead. Through experiments, we found that prediction models using contrastive learning have a better performance than other DL architectures. For example, our model increased the AUC of PD-1 and PD-L1 expressions by 8.41% and 8.57%, respectively, compared with ResNet-50, which shows that our model has stronger generalization and deep representation learning ability than other DL models. In addition, we introduced an extra data augmentation method called patch shuffle, which further enriched the feature patterns of the training CT images. This strategy improves the performance by 6.50% and 367 5.36% compared with the original augmentation strategies, which proves that this operation could help the model capture the invariance between the original image and the image shuffled on the patch unit, and thus enhance the model’s extraction of local representation

Furthermore, we introduced additional unlabeled CT images from patients with incomplete clinical information like PD-1 or PD-L1 expression during the self-supervised training phase. Training images are the cornerstones of the DL. In general, the more input images for model training, the more data patterns the model can accept, and the greater its generalization. However, we received several unlabeled medical images from patients with incomplete clinical information. Even though these images met the standard for network training, they were discarded owing to missing label information, which resulted in the waste of data resources. However, the contrastive learning we used is a self-supervised training method that trains the model without label information; therefore, we could reuse these unlabeled images that should have been discarded to increase the number of training images during this self-supervised training stage. We included these unlabeled CT images in the training set for the self-supervised training stage. The results in **Table 4** show that introducing additional unlabeled CT images significantly improved the performance of the prediction model (+4.21% for PD-1; +5.72% for PD-L1), which demonstrates that enriching the input patterns is an effective strategy for improving the model’s generalizability. This strategy is applicable to all self-supervised learning models and can effectively improve data utilization efficiency, providing a new method for future training data processing.

Our DL model simultaneously predicted the expression of PD-1 and PD-L1. The training phase of contrastive learning does not require label information, so the deep representation extracted by the convolution layers could be applied for the prediction of both PD-1 and PD-L1 expression. Therefore, the label information of PD-1 and PD-L1 expression is needed only to train the two full connection layers with fewer parameters, which could save training time and memory occupation to improve calculation efficiency. In addition, we used the joint loss function of the two full connection layers to update the predictor, which could strengthen the relevance of PD-1 and PD-L1 predictions and enrich the learning mode of the model. Compared with previous works on predicting the expression of a single protein (PD-1 or PD-L1), our model can obtain more comprehensive expression data from HCC patients, which is more conducive to the formulation of personalized treatment plans.

Although our model achieved promising results in the prediction of PD-1 and PD-L1 expression, there are still some limitations to our study. First, CLNet extracts deep representations of CT images by closing the distance between similar images, whereas useful representations are concentrated in the local area near the tumor lesion. While we used patch shuffle to enhance the learning of local representation, it is worth continuing to explore how to extract features from the local region by using better data augmentation methods or introducing attention mechanisms. Second, the data in this study were obtained from a single medical center, and the results need to be externally validated at other medical centers. Third, we only choose more DL network backbones for the contrastive learning. But the experiment illustrated the effectiveness of contrastive learning in the prediction of PD-1 and PD-L1 expression. So it is enough to only choose ResNet-50 as the backbone model.

For validating the effectiveness of our method for clinical use, we consider containing HCC patients’ CT images in other medical centers as an external validation set to test the performance of our model is a feasible strategy. Besides, we can observe whether including more CT images of HCC patients for model training could improve the model performance, which could also validate the generalization and clinical use of our model. We will positively try the above strategies in our future work.

## 5 Conclusion

In this study, we proposed a DL model for the noninvasive prediction of PD-1/PD-L1 expression in patients with HCC. Self-supervised contrastive learning and patch shuffle augmentation were used to help the model better extract deep representations of CT images. Based on the characteristics of self-supervised training of the model with unlabeled images, we introduced additional training images to improve the data utilization efficiency and the patterns of training images. Our DL model could simultaneously predict PD-1/PD-L1 expression, which is important for guiding the individualized treatment of patients with HCC. CLNet exhibited an AUC of 86.56% for PD-1 expression and an AUC of 83.93% for PD-L1 expression, performing better than other DL and machine learning models. The results of PD-1/PD-L1 expression prediction illustrate that our DL model may provide a new method for clinical decision-making in patients with HCC.

## Data availability statement

The original contributions presented in the study are included in the article/supplementary material. Further inquiries can be directed to the corresponding authors.

## Ethics statement

The studies involving human participants were reviewed and approved by Institutional Review Board (IRB) of West China Hospital. Written informed consent for participation was not required for this study in accordance with the national legislation and the institutional requirements.

## Author contributions

Conceptualization, LX, BS, and ML; Formal analysis, TX, YW, and XW; Data curation, YW, QL, FC, and QX; Methodology, TX, XC, MHL, and MY; Investigation, TX and YW; Project administration: QL, FC, QX, and FZ; Software, TX, XC, MHL, and MY; Supervision, BS, LX, and ML; Validation, XW, ML, and FZ; Visualization, XC, MHL, and MY; Writing, TX and YW. All authors contributed to the article and approved the submitted version.
